# Fluconazole impacts the extracellular matrix of fluconazole-susceptible and -resistant *Candida albicans* and *Candida glabrata* biofilms

**DOI:** 10.1080/20002297.2018.1476644

**Published:** 2018-06-04

**Authors:** Beatriz Helena Dias Panariello, Marlise I. Klein, Ewerton Garcia De Oliveira Mima, Ana Cláudia Pavarina

**Affiliations:** a Department of Cariology, Operative Dentistry and Dental Public Health, Indiana University– Purdue University Indianapolis, School of Dentistry, Indianapolis, IN, USA; b Department of Dental Materials and Prosthodontics, São Paulo State University (Unesp), School of Dentistry, Araraquara, São Paulo, Brazil

**Keywords:** Biofilm, *Candida albicans*, *Candida glabrata*, extracellular matrix, fluconazole, fluconazole-resistant

## Abstract

**Background**: Fluconazole (FLZ) is a drug commonly used for the treatment of *Candida* infections. However, β-glucans in the extracellular matrices (ECMs) hinder FLZ penetration into *Candida* biofilms, while extracellular DNA (eDNA) contributes to the biofilm architecture and resistance.

**Methods**: This study characterized biofilms of FLZ-sensitive (S) and -resistant (R) *Candida albicans* and *Candida glabrata* in the presence or absence of FLZ focusing on the ECM traits. Biofilms of *C. albicans* American Type Culture Collection (ATCC) 90028 (CaS), *C. albicans* ATCC 96901 (CaR), *C. glabrata* ATCC 2001 (CgS), and *C. glabrata* ATCC 200918 (CgR) were grown in RPMI medium with or without FLZ at 5× the minimum inhibitory concentration (37°C/48 h). Biofilms were assessed by colony-forming unit (CFU)/mL, biomass, and ECM components (alkali-soluble polysaccharides [ASP], water-soluble polysaccharides [WSP], eDNA, and proteins). Scanning electron microscopy (SEM) was also performed. Data were analyzed by parametric and nonparametric tests (*α*  =  0.05).

**Results**: In biofilms, FLZ reduced the CFU/mL of all strains (*p* < 0.001), except for CaS (*p* = 0.937). However, the ASP quantity in CaS was significantly reduced by FLZ (*p* = 0.034), while the drug had no effect on the ASP levels in other strains (*p* > 0.05). Total biomasses and WSP were significantly reduced by FLZ in the ECM of all yeasts (*p* < 0.001), but levels of eDNA and proteins were unaffected (*p* > 0.05). FLZ affected the cell morphology and biofilm structure by hindering hyphae formation in CaS and CaR biofilms, by decreasing the number of cells in CgS and CgR biofilms, and by yielding sparsely spaced cell agglomerates on the substrate.

**Conclusion**: FLZ impacts biofilms of *C. albicans* and *C. glabrata* as evident by reduced biomass. This reduced biomass coincided with lowered cell numbers and quantity of WSPs. Hyphal production by *C. albicans* was also reduced.

## Introduction

*Candida* species are commensal fungi of the oral cavity of healthy individuals that can become opportunistic pathogens in some situations, for example, when there are changes in the immune system, metabolic dysfunction, or in the elderly population [1–7]. Moreover, increased use of broad-spectrum antibiotics, cytotoxic chemotherapies, and transplantation also enhances the risk for infection by these opportunistic fungi [7]. These infections are known as candidiasis. *Candida albicans* is the main species associated with candidiasis, while *Candida glabrata* is one of the most predominant non-*albicans* species that has been linked to the development of oral infections [1–6]. It is an opportunistic pathogen in immunocompromised individuals [7,8], and it has an innate resistance to azoles [9,10]. *C. glabrata* differs from other pathogenic yeasts because of its phylogenetic position and haploid state with unconfirmed sexual cycle and diploid phase, contrary to *C. albicans* [11].

*Candida* infections are frequently related to the establishment of biofilms. Biofilms are composed by microbial cells that are attached to a substrate and surrounded by an extracellular matrix (ECM) [12]. The ECM is composed of secreted microbial and host-derived substances and cells lysis [12] and collaborates to the conservation of the biofilm architecture and the preservation of stable interactions between cell–cell, cell–surface, and the environment [13]. Although polysaccharides and protein are the most widely studied substances in biofilm ECMs, other molecules, such as nucleic acids, are important to their function [12].

Treatment of oral candidiasis includes the use of topical [14] and systemic [15] antifungal medication, such as fluconazole (FLZ). Systemic antifungal medication is usually prescribed for individuals with compromised overall health and those with recurrent infections [15]. An important aspect to be considered in therapy with topical or systemic antifungals is resistance to these drugs. Resistance to antifungal agents can be defined as the persistence or progression of infection after application of antimicrobial treatment [16,17]. Some studies have observed the emergence of resistant microorganisms during long-term or prophylactic treatment [18,19]. Intrinsic or primary resistance occurs when a microorganism has low susceptibility to a medication, before its exposure to the agent. Some *Candida* species possess intrinsic resistance to antifungal drugs, especially to FLZ [7,10]. In contrast, secondary resistance is one that can be developed by yeasts after long periods of exposure to antifungal drugs [16]. Thus, a major concern with *Candida* spp. biofilms is that their cells may have reduced susceptibility to azoles and polyenes [20,21].

Antifungal resistance of *Candida* biofilms is multifactorial and involves the stimulation of drug efflux pumps, the physiological state of cells, and the protection exerted by the ECM via β-glucans that bind to FLZ and amphotericin B [22], which limits diffusion of these antifungals through biofilms [23]. *C. albicans* biofilms that grow under constant flow produce higher quantities of ECM than statically cultured biofilms. Importantly, the former also have increased resistance to amphotericin B. This finding suggests that ECM presence has an association with resistance to amphotericin B in *Candida* species [24]. In the same study, the authors detected that the ECM of *C. albicans* GDH 2346 (NCYC 1467) contained carbohydrate, protein, hexosamine, phosphorus, and uronic acid. Nevertheless, the main constituent of the ECM of *C. albicans* was glucose (32%) [24]. In addition to β-glucan, it has been reported that extracellular DNA (eDNA) is also an important ECM constituent and one that promotes structural integrity of candidal biofilms [25]. Furthermore, nuclear magnetic resonance investigation verified interaction of ECM with FLZ, demonstrating a role in drug resistance [26].

The difficulties in establishing what ECM components are important in facilitating resistance to antifungals have been a challenge [27]. As such, enhanced knowledge of the assembly and function of ECM will help inform the future design of more effective therapies to control *Candida* pathogenesis and biofilm development. Here, we characterized the biofilms of FLZ-susceptible and -resistant *C. albicans* and *C. glabrata* strains in the presence or absence FLZ to evaluate the drug interference in the ECM of these microorganisms.

## Material and methods

### Yeasts

Four American Type Culture Collection (ATCC) *Candida* strains were used in this study: *C. albicans* ATCC 90028 (FLZ-susceptible; CaS), *C. albicans* ATCC 96901 (FLZ-resistant; CaR), *C. glabrata* ATCC 2001 (FLZ-susceptible; CgS), and *C. glabrata* ATCC 200918 (FLZ-resistant; CgR).

### Growth curves

**Figure 1.**
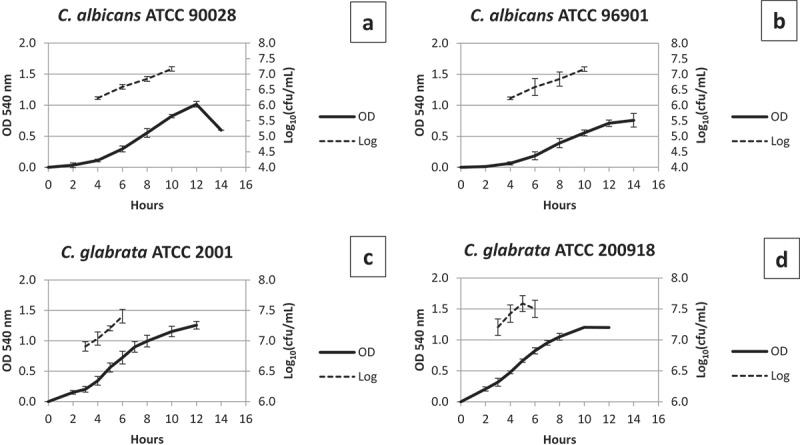
Growth curves of (A) *C. albicans* ATCC 90028 (CaS), (B) *C. albicans* ATCC 96901 (CaR), (C) *C. glabrata* ATCC 2001 (CgS), and (D) *C. glabrata* ATCC 200918 (CgR). In the mid-log phase, the mean ± standard deviation of OD_540__ __nm_ for CaS is 0.55 ± 0.08 and the Log_10_ CFU/mL is 6.8 ± 0.1. For CaR, the OD_540__ __nm_ is 0.39 ± 0.08 and the Log_10_ CFU/mL is 6.8 ± 0.3. For CgS, the OD_540__ __nm_ is 0.70 ± 0.08 and the Log_10_ CFU/mL is 7.4 ± 0.1. For CgR, the OD_540__ __nm_ is 0.65 ± 0.09 and the Log_10_ CFU/mL is 7.6 ± 0.2.

Growth curves were constructed based on optical density (OD) at 540 nm wavelength to ensure the reproducibility of the biofilm model. Growth curves were performed on three different occasions with three replicates. The number of colony-forming units (CFU) was determined at four-time points (Figure 1).

The microorganisms kept at −80°C were seeded onto Petri dishes with Sabourand dextrose agar (DIFCO, Detroit, MI) culture medium supplemented with chloramphenicol (50 mg/L) [28] and incubated at 37°C for 48 h. Then, five colonies of each microorganism were added separately to tubes containing Yeast Nitrogen Base medium (YNB – DIFCO, Detroit, MI) supplemented with 100 mM of glucose [28] and these pre-inocula were incubated at 37°C for 16 h. Subsequently, the pre-inocula were diluted with fresh YNB medium plus 100 mM glucose (1:20 dilution for *C. albicans* strains and 1:10 for *C. glabrata* strains) to form the inocula, and the OD_540 nm_ and CFU were determined every 2 h in a total of 16 h for each strain. Biofilms were generated using mid-log growth phase cells (Figure 1).

### Susceptibility testing and FLZ concentrations for biofilm formation

The CLSI M27-A3 broth microdilution susceptibility method [29] was performed with modifications to examine the minimal inhibitory concentrations (MICs) of FLZ against planktonic FLZ-susceptible and -resistant *C. albicans* and *C. glabrata* strains. FLZ powder (F8929, Sigma-Aldrich, St. Louis, MO) was dissolved in sterile ultrapure water [30]. As a positive control, a 100 µL of 2× concentrated RPMI 1640 buffered to pH 7.0 with 0.165 M (3-(*N*-morpholino)propanesulfonic acid) (MOPS) plus a 100 µL of sterile ultrapure water was used (no cells nor antifungal agent). As a negative control, only cell suspensions were tested without the antifungal agent. Serial twofold dilutions of FLZ (range: 0.125–512 μg/mL) in RPMI 1640 medium buffered to pH 7.0 with 0.165 M of MOPS were inoculated in 96-well plates with each microorganism suspension adjusted to achieve a final inoculum concentration of 0.5 × 10^3^–2.5 × 10^3^ CFU/mL based on the growth curves (Figure 1). The plates were incubated at 37°C and observed for the presence or absence of growth at 24 h. In addition to visual endpoint readings, the OD of each plate well was measured at 562 nm after 24 h of incubation. MICs in spectrophotometer were based on the density of the growth control and were considered as the lowest drug concentrations that resulted in minimum 90% decrease in growth related to the drug-free growth control [31,32]. MICs were recorded in duplicate on three separate occasions. As cells in biofilms are more resistant to drugs [33], the concentrations of 5× and 10× were tested during 48 h biofilm formation [34]. The 5× MIC concentration was chosen because 10× MIC almost completely inhibited the formation of biofilm (data not shown), and this was not the goal of the study since the inhibition of biofilm does not allow the evaluation of FLZ effects on biofilms’ ECMs. Thus, biofilms were generated in the presence of FLZ at 5× MIC.

### Biofilm formation and processing

Biofilm formation and processing for Log_10_ CFU/mL, total biomass, insoluble biomass, proteins (from the insoluble part of the biofilm and from the supernatant), alkali-soluble polysaccharide (ASP), water-soluble polysaccharide (WSP), and eDNA were performed according to the methodology described by Panariello et al. [35]. Briefly, biofilms of CaS, CaR, CgS, and CgR were developed for 48 h in RPMI buffered with MOPS (pH 7.0). Biofilms (48 h) were washed twice with 0.89% NaCl and detached by individually scratching the wells with a pipette tip and 2 mL 0.89% NaCl. Then, biofilms were separated for Log_10_ (CFU/mL) determination (0.1 mL) and for total biomass determination [36] (0.1 mL). After that, biofilms were vortexed, centrifuged (5,500×*g*/10 min/4°C) and the supernatant containing the soluble part of the matrix was separated from the pellet, which contained the insoluble part of the matrix. The supernatant was divided for the quantification of WSPs (1 mL) [37], eDNA (0.650 mL) [38], and matrix protein in the soluble portion (0.150 mL) [39]. The pellet was washed twice and resuspended in sterile ultrapure water; then, it was separated for the quantification of insoluble biomass (0.8 mL), protein from the insoluble portion (0.05 mL) [39], and ASPs (0.95 mL) [37].

### Scanning electron microscopy

Biofilms were grown on polystyrene coupons placed in 24-well culture plates [40]. Prior to use, coupons were sterilized by microwave treatment for 3 min at 650 W [41] and dried in a flow chamber with UV light for 30 min. After 48 h of biofilms growth, the medium was removed, and the wells were washed twice with 1 mL of sterile 0.89% NaCl. Next, the biofilm samples were fixed with 2.5% glutaraldehyde (60 min/room temperature), washed twice with sterile 0.89% NaCl, and dehydrated. The dehydration process was performed with series of washes with ethanol 70 and 90% for 60 min each, followed by five washes of 30 min with absolute ethanol. The samples were stored in a desiccator with silica for 7 days to guarantee moisture-free samples. Then, the samples were fixed in aluminum stubs, sputter coated with gold, and observed with a JEOL JSM-6610LV Scanning Electron Microscope, using magnifications of 160×, 600×, and 1,200×.

### Statistical analyses

Normal distribution of data was verified by the Shapiro–Wilk test and homogeneity of variance was checked by the Levene test (*α* = 0.05). The quantitative data of CFU/mL, biomass, proteins from the insoluble portion and ECM components were statistically analyzed by two-way analysis of variance (ANOVA) considering the presence or absence of FLZ and the different strains (CaS, CaR, CgS, CgS). For multiple comparisons, the Tukey *post hoc* test was applied for homoscedastic data and the Games-Howell *post hoc* test for heteroscedastic data (*α* = 0.05). When a postulation of normality was not encountered, data were ranked and a nonparametric analysis (ANOVA on ranks) was applied (*α* = 0.05). The analyses were done in the software SPSS (IBM® SPSS® Statistics, version 20, Chicago, IL).

## Results

### Susceptibility testing and FLZ concentrations for biofilm formation

The MIC_90_ for planktonic cells were CaS = 16 µg/mL, CaR = 256 µg/mL, CgS = 8 µg/mL, and CgR = 256 µg/mL. However, the MIC_90_ concentration found for planktonic cells of CaS is not typical for sensitive strains. As cited before, cells in biofilms are more resistant than cells in planktonic cultures; thus, for biofilm formation, 5× MIC was applied: CaS = 80 µg/mL, CaR = 1,280 µg/mL, CgS = 40 µg/mL, and CgR = 1,280 µg/mL.

### Biofilm and ECM characterization

**Figure 2.**
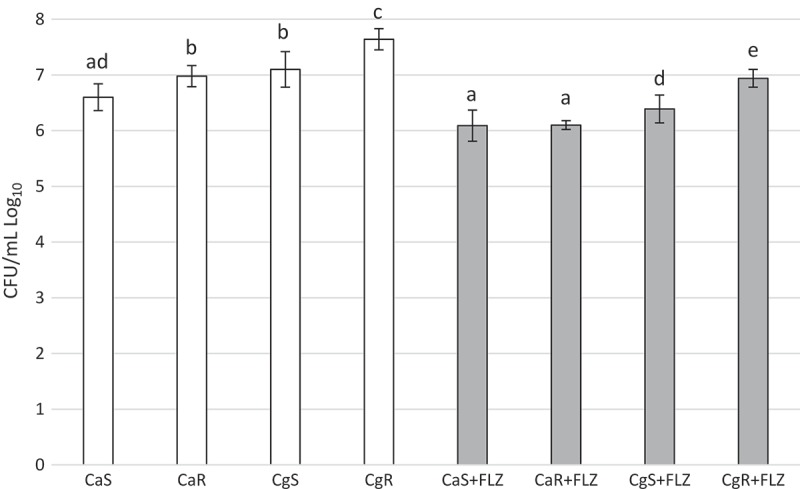
Mean values and standard deviations of Log_10_ (CFU/mL) of CaS, CaR, CgS, and CgR in the absence (white bars) or in the presence (gray bars) of fluconazole (FLZ). Bar charts were applied for normal data submitted to parametric analysis (ANOVA). Equal letters designate statistical correspondence (*p* < 0.05).

For Log_10_ (CFU/mL), there was a significant interaction between the factors ‘FLZ’ and ‘strains’ (*p* < 0.001). FLZ significantly reduced (*p* < 0.001) the Log_10_ (CFU/mL) values of CaR (reduction of 0.88 log), CgS (reduction of 0.71 log), and CgR (reduction of 0.70 log). In contrast, the statistics pointed out that the reduction of 0.67 log caused by FLZ on CaS biofilms was not significant (*p* = 0.937). Moreover, CaS presented the lowest CFU/mL of all the strains in the absence of FLZ (*p* < 0.001). Mean and standard deviations of Log_10_ (CFU/mL) are represented in Figure 2.

**Figure 3.**
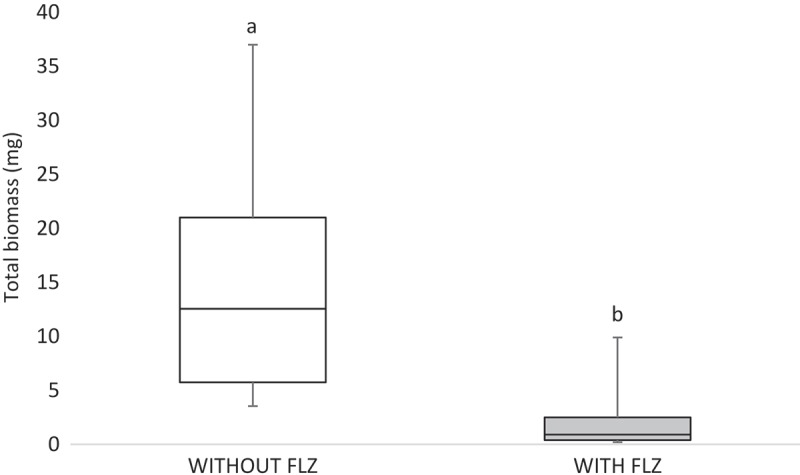
Box-plot of total biomass (mg) without (white bar) and with FLZ (gray bar). The box-plot shows the median (dash), the first and third quartiles (outer edges of box), and the highest and maximum values (error bars). A nonparametric analysis (ANOVA on rank) was applied. Equal letters designate statistical correspondence (*p* > 0.05).

**Figure 4.**
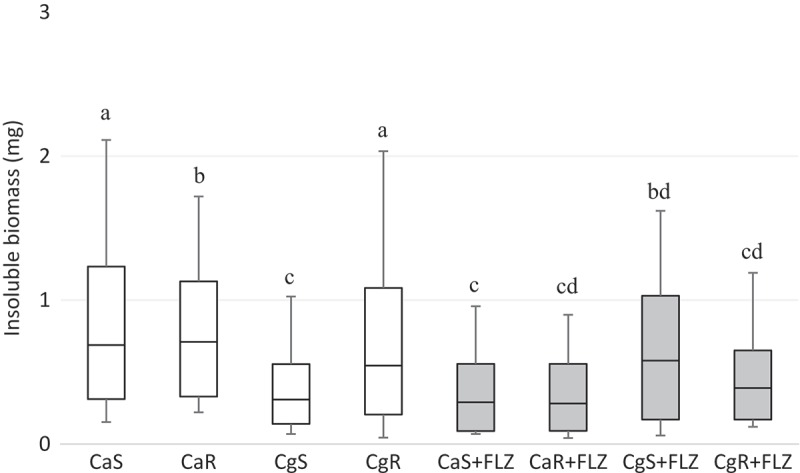
Box-plot of insoluble biomass (mg) in CaS, CaR, CgS, and CgR in the absence (white boxes) or in the presence (gray boxes) of FLZ. The box-plot shows the median (dash), the first and third quartiles (outer edges of box), and the highest and maximum values (error bars). A nonparametric analysis (ANOVA on rank) was applied. Equal letters designate statistical correspondence (*p* > 0.05).

The total biomass analysis showed that the factor ‘FLZ’ significantly interacted with the results (*p* < 0.001), reducing the biomasses of all the strains (Figure 3). For the insoluble biomass, data pointed to an interaction between the factors ‘FLZ’ and ‘strains’ (*p* < 0.001). The presence of FLZ significantly reduced the insoluble biomass of CaS (*p* = 0.038), CaR (*p* < 0.001), and CgR (*p* < 0.001). In contrast, CgS showed higher insoluble biomass in the presence of FLZ when compared to CgS in the absence of FLZ (*p* < 0.001). Mean and standard deviations of insoluble biomasses are given in Figure 4.

**Figure 5.**
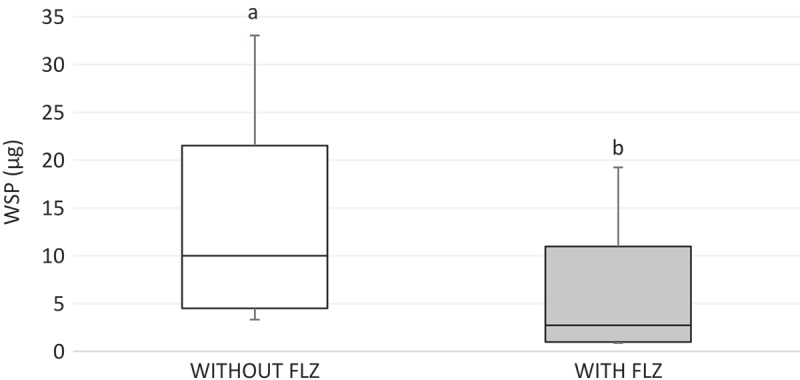
Box-plot of water-soluble polysaccharides-WSP amounts (μg) without (white bar) and with FLZ (gray bar). The box-plot shows the median (dash), the first and third quartiles (outer edges of box), and the highest and maximum values (error bars). A nonparametric analysis (ANOVA on rank) was applied. Equal letters designate statistical correspondence (*p* > 0.05).

**Figure 6.**
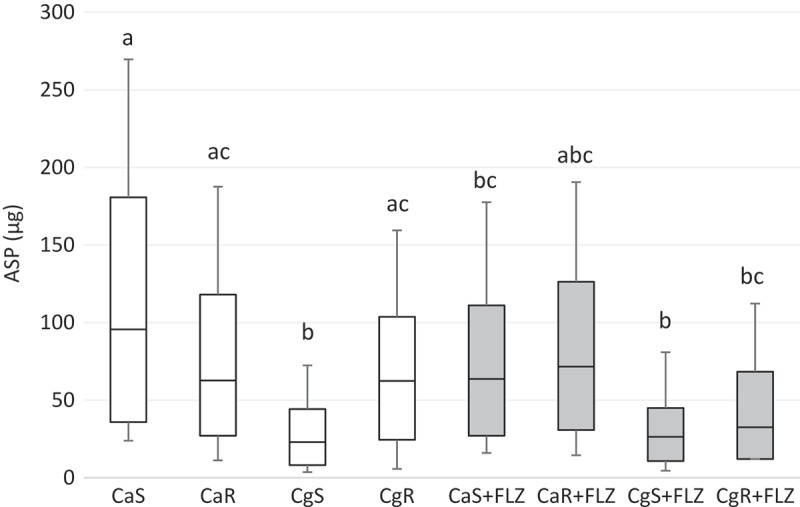
Box-plot of alkali-soluble polysaccharides-ASP amounts (μg) in CaS, CaR, CgS, and CgR in the absence (white boxes) or in the presence (gray boxes) of FLZ. The box-plot shows the median (dash), the first and third quartiles (outer edges of box), and the highest and maximum values (error bars). A nonparametric analysis (ANOVA on rank) was applied. Equal letters designate statistical correspondence (*p* < 0.05).

For WSP data, the analysis showed that the only factor that influenced the quantity of WSP was ‘FLZ’ (*p* < 0.001), reducing this component in the ECM of all the evaluated strains (Figure 5). For ASP data, a significant interaction between the factors ‘FLZ’ and ‘strains’ (*p* = 0.002) was found. Statistics demonstrated that FLZ significantly reduced the ASP amount in CaS (*p* = 0.034). However, for the other strains, FLZ did not alter the ASP amounts (*p* > 0.200). Moreover, CgS produced significantly smaller amounts of ASP than the other evaluated strains (*p* = 0.008). Mean and standard deviations of ASP are presented in Figure 6.

**Figure 7.**
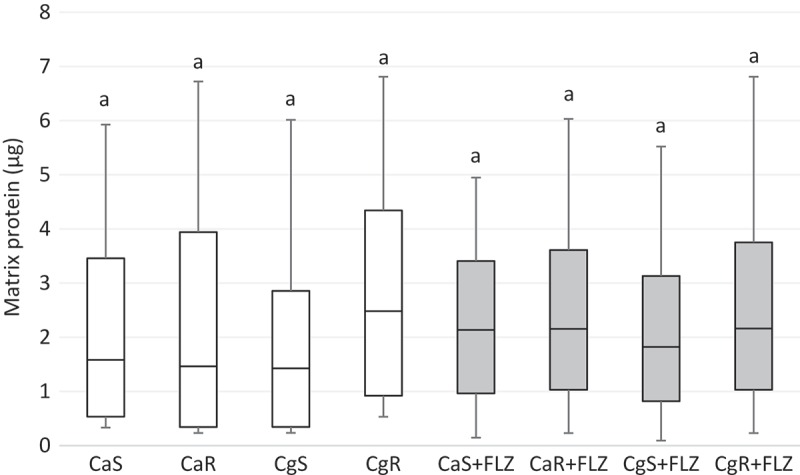
Box-plot of matrix protein amounts (μg) in CaS, CaR, CgS, and CgR in the absence (white boxes) or in the presence (gray boxes) of FLZ. The box-plot shows the median (dash), the first and third quartiles (outer edges of box), and the highest and maximum values (error bars). A nonparametric analysis (ANOVA on rank) was applied. Equal letters designate statistical correspondence (*p* < 0.05).

Two-way analysis for matrix protein data showed that the factor 'strain' was significant (*p*< 0.001). However, *post hoc* tests showed no statistical differences in the quantity of matrix proteins between the strains in the presence or the absence of FLZ (*p* > 0.05) (Figure 7). Protein data from the insoluble portion showed that there was no significance of factors ‘FLZ’ (*p* = 0.124), ‘strain’ (*p* = 0.124), and the interaction between ‘FLZ’ and ‘strain’ (*p* *=* 0.573). Thus, no *post-hoc* tests were performed. Consequently, protein amounts from the insoluble portion were not affected by FLZ, and the type of the strain did not influence the quantities of this component.

For eDNA, data revealed that there was no significance of the factors ‘FLZ’ (*p* = 0.936), ‘strain’ (*p* = 0.431), and between the factors ‘FLZ’ and ‘strains’ (*p* = 0.974). Therefore, eDNA quantities were not affected by FLZ and were the same independent of the type strain. As there was no statistical significance of any factor or interaction for protein from the insoluble portion and for the eDNA data, mean and standard deviations from the quantification of these components are shown in Table 1.

**Table 1.** Proteins from the insoluble portion (µg) and eDNA (µg) in sensitive and resistant *C. albicans* and *C. glabrata* to fluconazole (FLZ).

	Proteins from the insoluble portion (µg)	eDNA (µg)
CaS	1.16	28.32
	(0.77)	(8.65)
CaR	1.35	34.98
	(1.07)	(16.50)
CgS	1.25	25.41
	(0.96)	(11.36)
CgR	1.52	25.59
	(0.74)	(10.76)
CaS + FLZ	1.16	36.14
	(0.23)	(8.72)
CaR + FLZ	1.29	28.27
	(0.47)	(10.94)
CgS + FLZ	1.07	28.39
	(0.33)	(11.53)
CgR + FLZ	1.29	20.97
	(0.47)	(6.58)

Two-way ANOVA revealed that there was no significance of the factors ‘FLZ’, ‘strain’, and between the factors ‘FLZ’ and strains for protein and eDNA, meaning there was no statistical difference between the conditions depicted for each of the components.

### Biofilm structure

**Figure 8.**
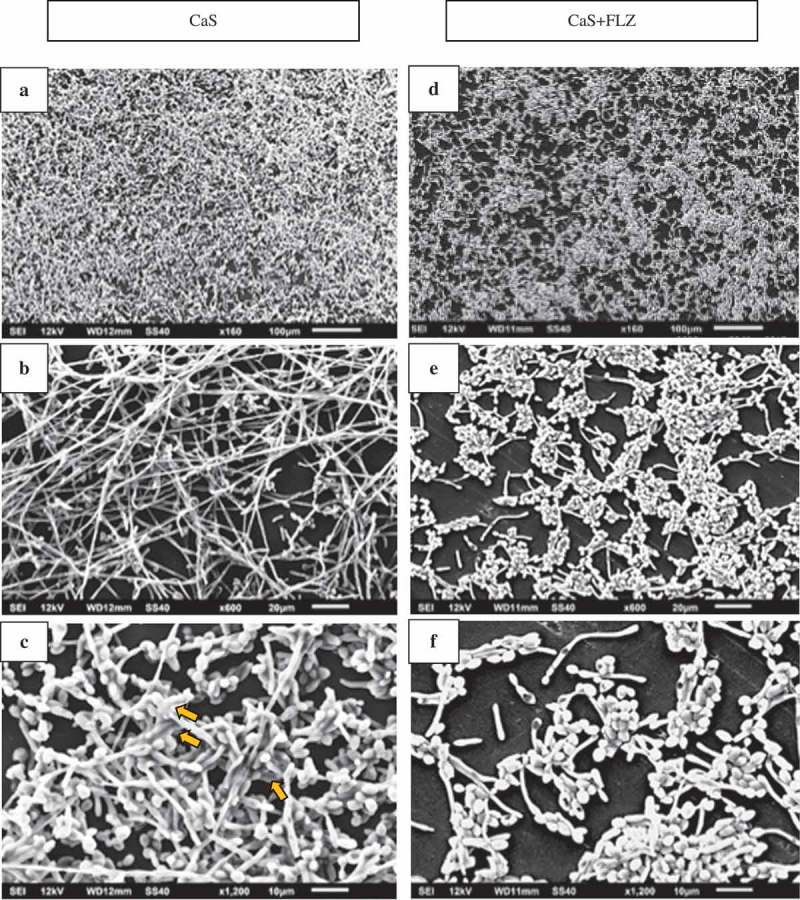
Scanning electron microscopy (SEM) images of CaS (A,B,C) and CaS + FLZ (D,E,F). FLZ (80 µg/mL) reduced the size of the hyphae (D,E,F). A closer look at the cells structures shows that without FLZ, it is possible to see the cells embedded in the ECM, as shown by the arrow (C).

Scanning electron microscopy (SEM) was performed to examine the overall biofilm structure of the different yeasts in the presence and absence of FLZ. Special attention was given to cell morphology and spatial organization on the substrate, as well as on ECM covering and/or linking cells in these biofilms. Figure 8 shows the SEM images of CaS (Figure 8(A–C)) and CaS + FLZ (Figure 8(D–F)), demonstrating that FLZ reduced the size of hyphae. A closer look at the structures shows that, without FLZ, cells were embedded in the ECM, as shown by the arrow (Figure 8(C)).

**Figure 9.**
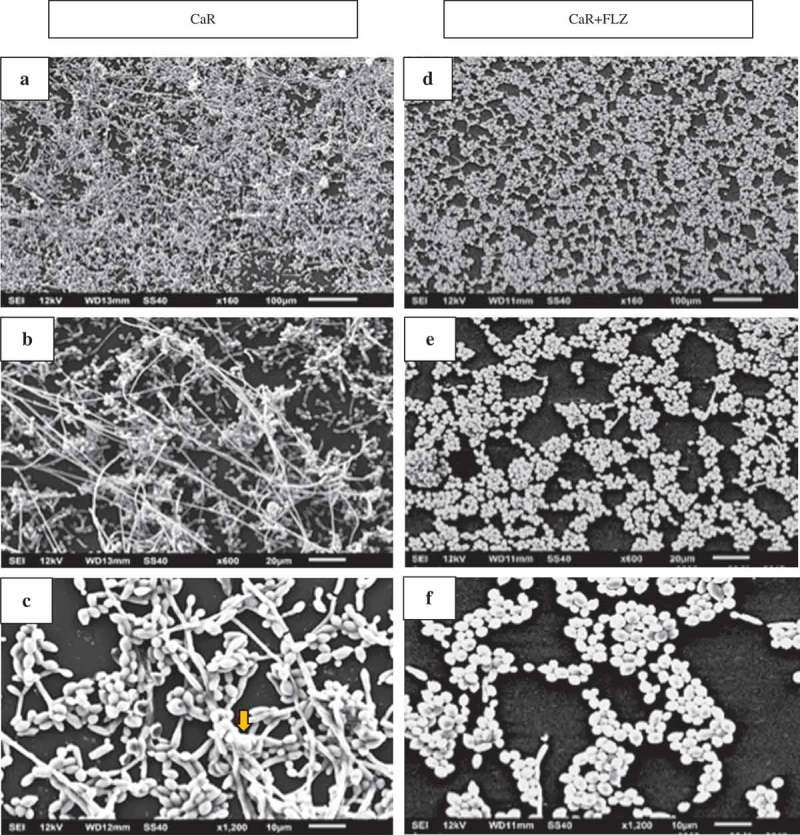
SEM images of CaR (A,B,C) and CaR + FLZ (D,E,F). The CaR biofilm possesses large amounts of yeast cells and elongated hyphae structures (B,C). Image C shows the union of cells within biofilms through their extracellular matrices, as exemplified by the arrow. FLZ (1,280 µg/mL) reduced the number and size of hyphae, remaining mostly yeast cells (D,E,F).

Figure 9 shows the SEM images of biofilms formed by CaR (Figure 9(A–C)) and CaR + FLZ (Figure 9(D–F)). The CaR biofilm possessed large amounts of yeast cells and elongated hyphae structures (Figure 9(A–C)). Figure 9(C) shows the union of cells within biofilms through their ECMs, as exemplified by the arrow. FLZ reduced the size and number of hyphae; remaining cells were mostly cells with yeast morphology (Figure 9(C–E)).

**Figure 10.**
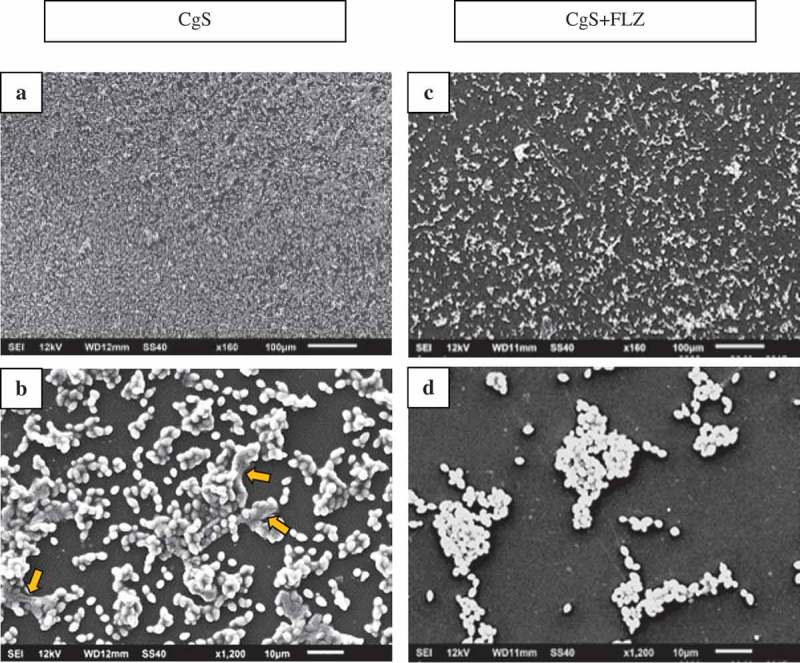
SEM images of CgS (A,B) and CgS + FLZ (C,D). CgS biofilm is composed by yeast cells (A) linked by ECM (B). The presence of FLZ (40 µg/mL) reduced the number of cells (C) and the ECM (D).

Figure 10 shows the SEM images of biofilms formed by CgS (Figure 10(A,B)) and CgS + FLZ (Figure 10(C,D)). The CgS biofilm was composed of yeast cells (Figure 10(A)) linked by ECM (Figure 10(B)). The presence of FLZ reduced the number of cells (Figure 10(C)) and the ECM (Figure 10(D)).

**Figure 11.**
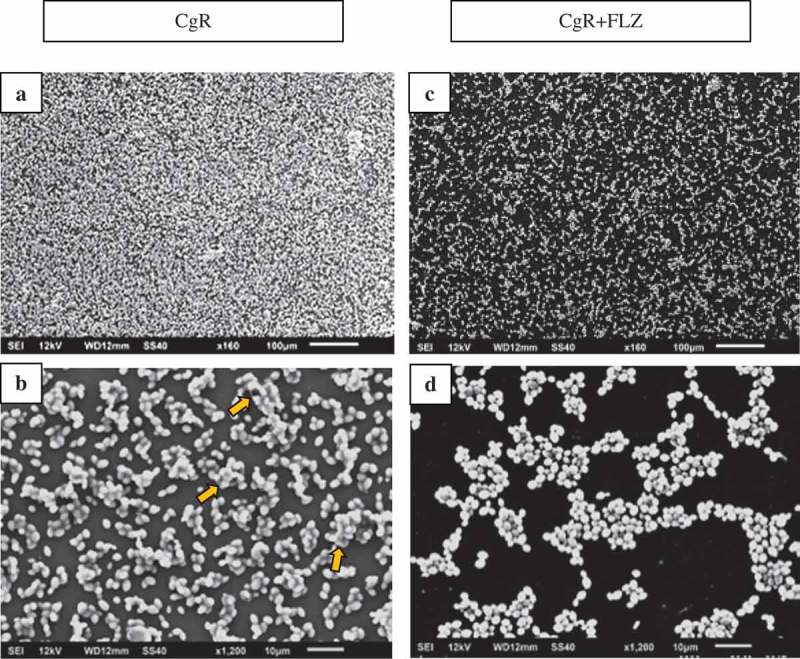
SEM images of CgR (A,B) and CgR + FLZ (C,D). CgR biofilm presents a high-quantity yeast cells (A) and clusters interconnected by ECM, exemplified by arrows (B). The presence of FLZ (1,280 µg/mL) reduced the number of cells (C) and the ECM (D).

Figure 11 shows the SEM images of biofilms formed by CgR (Figure 11(A,B)) and CgR + FLZ (Figure 11(C,D)). The CgR biofilm presented a high quantity of yeast cells (Figure 11(A)) and clusters interconnected by ECM (Figure 11(B)). The presence of FLZ reduced the number of cells (Figure 11(C)) and the ECM (Figure 11(D)).

## Discussion

FLZ is often a chosen treatment for *Candida* infections because of its low cost and availability for oral administration [42]. This drug prevents the biosynthesis of ergosterol through the cytochrome P450 enzyme 14-α demethylase, which catalyzes the conversion of lanosterol to ergosterol [43]. The reduction of ergosterol changes the fluidity of the membrane and the activity of numerous membrane-bound enzymes, hindering fungal growth and replication [43]. However, FLZ-resistance is rising in *Candida* species [42,44,45,46], requiring a better understanding of the action of this drug in *Candida* biofilms. Due to the mechanism of action of FLZ, variations in the architecture of the ECM may happen during *Candida* biofilm formation in presence of this medication [34,47]. Here, we characterized the biofilms formed by FLZ-susceptible and -resistant *C. albicans* and *C. glabrata* strains in presence or absence FLZ to evaluate the drug interference in the ECM of these microorganisms.

Surprisingly, no significant reduction in CaS counts was observed after exposure to 5× MIC doses of FLZ (Figure 2), and the images showed that FLZ reduced the size of hyphae in the strain (Figure 8(C–E)). However, the growth of CaR biofilms in the presence or absence of FLZ showed that the drug significantly reduced its population (Log_10_ CFU/mL) (Figure 2). In addition, SEM images showed that FLZ reduced hyphae formation in CaR (Figure 9(D–F)). This result corroborated a previous study that observed that FLZ has a direct inhibitory effect on hyphal formation [48]. Since FLZ interferes with the ergosterol pathway and ergosterol is necessary for hyphae formation, the presence of FLZ inhibits the transition from yeast to hypha [48], even in a culture medium that promotes *C. albicans* filamentous morphology. Thus, taking into consideration that hypha is the invasive form of *C. albicans* [49], the reduction of its size in both CaS and CaR is an important result in the decrease of the virulence of this strain. In addition, FLZ significantly reduced the CFU/mL in CgS and CgR (Figure 2). These results were confirmed by the SEM images of CgS (Figure 10(D,E)) and CgR (Figure 11(D,E)), which showed that fewer cells and cell agglomerates were more sparsely distributed on the substrate. It has been reported that *C. glabrata* has a decreased intrinsic susceptibility to FLZ and other classes of azole antifungals [42,50,51]. Nevertheless, the results of the present study showed that biofilm formation with 40 µg/mL of FLZ for CgS and 1,280 µg/mL of FLZ for CgR can reduce the cell viability of these microorganisms. Therefore, the log reduction values for all strains were very close (CaS: reduction of 0.67 log, CaR: reduction of 0.88 log, CgS: reduction of 0.71 log, and CgR: reduction of 0.70 log).

An important finding of this study was that FLZ acted on the ECM of all the yeasts by reducing the production of WSPs (Figure 5). *Candida* biofilm matrix has α-1,2 branched and α-1,6 mannans (WSPs) associated with β-1,6 glucans (ASPs), constituting a mannan–glucan complex (MGCx) [26]. Considering that interactions of ECM polysaccharides are required for biofilm antifungal resistance [52], the reduction on WSP amounts in the ECM of the biofilms in the presence of FLZ might alter the constitution of the MGCx, leading to a higher susceptibility of the biofilms to antifungal therapy. Another notable finding of this study was that FLZ caused an important antibiofilm activity by causing a significant reduction in the total biomasses of all the yeasts studied. Cells and ECM components compose the biomasses. Thus, the significant reduction of the biomasses could have occurred because of the decrease of WSP, the reduction of the hyphae size in CaS (Figure 8(D–F)) and CaR (Figure 9(D–F)), and the decrease in cells in CgS (Figure 10(C,D)) and CgR (Figure 11(C,D)).

In addition, FLZ significantly reduced the ASPs content only in the ECM of the CaS strain biofilm (Figure 6). The ASPs, such as β-1,3 glucans, sequestrate antifungals preventing them from diffusing through the ECMs of *C. albicans* [22,52–55] and non-*albicans* biofilms [56]. It has been demonstrated that β-1,3 glucan has a role in antifungal resistance as it binds to FLZ avoiding this drug to reach its targets [22,52–56]. Thus, ASP’s significant reduction in the ECM of CaS biofilms demonstrates that the biofilms of this specific strain become more vulnerable when FLZ is present. Moreover, although ASPs can bind to FLZ, when 80 μg/mL of FLZ is present during CaS biofilm formation, the drug can evade this effect and cause ASP reduction.

The insoluble content of the biofilm (i.e. ASP and cells, which contain the proteins) constitutes the insoluble biomass. The results showed that in the presence of FLZ, there was a significant reduction in the insoluble biomass of CaS, CaR, and CgR (Figure 4) probably caused by the reduction of hyphae and blastopores, and by the numerical decrease in the ASP content. However, CgS showed a significant increase in the insoluble biomass in presence of FLZ. Variations in the ECM might happen when ions are available [57]. Possibly, ions from FLZ (C_13_H_12_F_2_N_6_O) might have interacted with carboxylic acid groups of the matrix to produce grids of macromolecules with higher viscosity resulting in a stickier polysaccharide matrix [57].

FLZ caused no effects on eDNA, proteins (from the insoluble part of the biofilm), and matrix protein amounts in all the strains evaluated. The production of eDNA has been associated to hyphal growth [58]. Nevertheless, the present study demonstrated that even in the absence of hyphae (e.g. in *C. glabrata* biofilms), all biofilms released comparable amounts of eDNA both in presence or absence of FLZ. This result corroborates to a previous finding that observed that a strain defective in hyphae formation (Δ/Δ efg1) was capable of producing eDNA similar to its parental strains that formed hyphae [35]. In *C. albicans*, eDNA contributes to the preservation and stability of mature biofilms, but not to their establishment [24,25], while the recognized role of some proteins is linked to metabolism [26]. In addition, eDNA performs as a regulator of biofilm cell antifungal resistance against echinocandins and amphotericin B but does not seem to significantly contribute to FLZ resistance [59]. This finding corroborates with the results found here, since FLZ did neither affect the production of eDNA nor the production of proteins.

The present study showed that WSPs and biomasses are directly affected by the presence of FLZ during biofilm formation in the FLZ-susceptible and -resistant *C. albicans* and *C. glabrata* strains studied, while eDNA and proteins were not altered by the presence of FLZ, independent of the strain. Thus, FLZ impacts biofilms of *C. albicans* and *C. glabrata* as evident by reduced biomass. The reduced biomass coincided with lowered cell numbers and the quantity of WSPs. Hyphal production by *C. albicans* was also reduced. The reduction of WSP and total biomasses may be related to biofilms susceptibility to therapeutics and virulence, making those growing in FLZ more susceptible and potentially less virulent. However, as FLZ is fungistatic, there is a chance for the development of acquired resistance during the treatment with this antifungal agent [46]. Moreover, 5× MIC concentrations of the drug during biofilm formation did not affect eDNA and proteins. Therefore, it is important to consider alternative ways to disorganize the ECMs to get a better diffusion of the drug through the biofilms to reach the cells.
